# Loss of CAMK2G affects intrinsic and motor behavior but has minimal impact on cognitive behavior

**DOI:** 10.3389/fnins.2022.1086994

**Published:** 2023-01-06

**Authors:** Pomme M. F. Rigter, Charlotte de Konink, Geeske M. van Woerden

**Affiliations:** ^1^Department of Clinical Genetics, Erasmus Medical Center, Rotterdam, Netherlands; ^2^Erfelijke Neuro-Cognitieve Ontwikkelingsstoornissen, Expertise Centre for Neurodevelopmental Disorders, Erasmus Medical Center, Rotterdam, Netherlands; ^3^Department of Neuroscience, Erasmus Medical Center, Rotterdam, Netherlands

**Keywords:** *Camk2g* knockout, behavior, plasticity, isoforms, neurodevelopment

## Abstract

**Introduction:**

The gamma subunit of calcium/calmodulin-dependent protein kinase 2 (CAMK2G) is expressed throughout the brain and is associated with neurodevelopmental disorders. Research on the role of CAMK2G is limited and attributes different functions to specific cell types.

**Methods:**

To further expand on the role of CAMK2G in brain functioning, we performed extensive phenotypic characterization of a *Camk2g* knockout mouse.

**Results:**

We found different CAMK2G isoforms that show a distinct spatial expression pattern in the brain. Additionally, based on our behavioral characterization, we conclude that CAMK2G plays a minor role in hippocampus-dependent learning and synaptic plasticity. Rather, we show that CAMK2G is required for motor function and that the loss of CAMK2G results in impaired nest-building and marble burying behavior, which are innate behaviors that are associated with impaired neurodevelopment.

**Discussion:**

Taken together, our results provide evidence for a unique function of this specific CAMK2 isozyme in the brain and further support the role of CAMK2G in neurodevelopment.

## Introduction

The calcium/calmodulin-dependent protein kinase 2 (CAMK2) family consists of four different isozymes, CAMK2A, CAMK2B, CAMK2G, and CAMK2D, transcribed from individual genes but with high homology as they all consist of the same domains. Of these domains, the linker domain between the regulatory and association domain shows substantial variation between members (Sloutsky and Stratton, 2021). All four isozymes are expressed in the brain, though at different expression levels (Tobimatsu and Fujisawa, 1989; Bayer et al., 1999; Murray et al., 2003). The role of CAMK2A and CAMK2B in brain functioning has been studied extensively using different mouse models, revealing their critical role in learning and synaptic plasticity (Silva et al., 1992a,b; Giese et al., 1998; Elgersma et al., 2002; van Woerden et al., 2009; Borgesius et al., 2011; Achterberg et al., 2014). This is accentuated by the discovery of mutations in the corresponding genes of patients suffering from neurodevelopmental disorders with intellectual disability (Küry et al., 2017; Akita et al., 2018; Chia et al., 2018; Rizzi et al., 2020; Heiman et al., 2021; Proietti Onori and van Woerden, 2021). Studying the role of CAMK2G in the brain has only recently become a focus of interest, amongst others due to the identification of patients with neurodevelopmental disorders carrying a mutation in the *CAMK2G* gene (de Ligt et al., 2012; Proietti Onori et al., 2018), highlighting the function of CAMK2G in normal brain functioning.

CAMK2G expression can be detected by embryonic day 11.5 (E11.5) in mice, and it is expressed throughout the brain in excitatory neurons, interneurons, and astrocytes (Tobimatsu and Fujisawa, 1989; Sakagami and Kondo, 1993; Bayer et al., 1999; Vallano et al., 2000; Batiuk et al., 2020; Huntley et al., 2020; Kozareva et al., 2021). The highest expression is observed in interneurons, and both global and interneuron-specific loss of CAMK2G results in impaired cognition and plasticity (Cohen et al., 2016, 2018; He et al., 2021, 2022). Different splice variants are expressed by the *Camk2g* gene, one of which contains a nuclear localization signal (NLS) in its variable domain. This specific isoform is shown to shuttle calcium-bound calmodulin to the nucleus in excitatory neurons, potentially playing a role in excitation–transcription coupling (Ma et al., 2014; Cohen et al., 2016). However, in interneurons, CAMK2G is not involved in calmodulin shuttling to the nucleus (Cohen et al., 2016). Additionally, knockdown of *Camk2g* was found to cause abnormal neurite growth in primary neurons, which could be normalized by re-expressing both a CAMK2G isoform with as well as without the NLS (Proietti Onori et al., 2018). Together, these results show that more research is required to get full insight into the role of CAMK2G in neurons and more broadly, in the brain.

Similar to mice, in humans *CAMK2G* is one of the main CAMK2 isozymes expressed during early neurodevelopment (Proietti Onori et al., 2018). The finding that a mutation in this gene causes a neurodevelopmental disorder (Proietti Onori et al., 2018) suggests that CAMK2G plays an important role in neurodevelopment. Patients carrying mutations in *CAMK2G* show, besides intellectual disability, a spectrum of phenotypic traits (Proietti Onori et al., 2018). To provide further insight into the role of CAMK2G in normal brain function, we obtained global *Camk2g* knockout mice and performed an extensive phenotypic characterization. We tested cognitive behavior but also other forms of behavior as patients with *CAMK2G* missense mutations suffer from severe intellectual disability and autism spectrum disorder but also impaired motor skills, hypotonia during infancy, and atypical body growth development (Proietti Onori et al., 2018). Our results indicate that in contrast to CAMK2A and CAMK2B, CAMK2G is not essential for hippocampus-dependent learning or synaptic plasticity, which contradicts previous *Camk2g* knockout mice studies (Cohen et al., 2018; He et al., 2021). Instead, we found that CAMK2G plays a role in motor and innate behavior, further supporting the role of CAMK2G in neurodevelopment.

## Materials and methods

### Mouse line

We obtained the *Camk2g* knockout mouse by ordering frozen sperm heterozygous for the MGI allele Camk2g^*TM*1*a(EUCOMM)Wtsi*^ in a C57BL/6N background from the European Conditional Mouse Mutagenesis Program (EUCOMM). A super ovulating female C57BL/6J mouse was inseminated by IVF, followed by the rederivation of the fertilized eggs to a surrogate C57BL/6J mom. To obtain the experimental groups, heterozygous Camk2g^*TM*1*a(EUCOMM)Wtsi*^ mice were crossed and the genotype was determined by PCR. For the experiments, we used male and female adult mice (>8 weeks old). They were group-housed at 22 ± 2°C, except for the nest-building test for which they had to be single-caged, and had food and water available *ad libitum* in a 12/12-h light/dark cycle. Experiments were executed during the light phase by an experimenter blind to the genotypes. All experiments with animals were conducted in accordance with the European Commission Council Directive 2010/63/EU (CCD project license AVD101002017893), and all described experiments and protocols were ethically approved by an independent review board of the Erasmus MC.

### Western blot on isolated brain regions

Animals were anesthetized by isoflurane and decapitated. The brain was quickly removed from the skull and the cerebellum and brainstem were separated. The cerebrum was turned upside down, and the hypothalamus was isolated. Next, it was cut in half coronally, and from the anterior part, the striatum was isolated. The leftover tissue, predominantly the anterior cortex, but without the olfactory bulb, was labeled “forebrain.” From the posterior cerebrum, the cortex was isolated and collected. Now the oval-shaped hippocampus was visible and isolated. The leftover tissue, predominantly the thalamus, was labeled “diencephalon.” As soon as the regions were collected in Eppendorf tubes, they were snap-frozen in liquid nitrogen.

Samples were sonicated in lysis buffer (0.1M Tris–HCl of pH 6.8, 4% SDS) with 1:20 protease inhibitor cocktail (P8340, Sigma, St Louis, MO, United States) and 1:40 phosphatase inhibitor cocktail 2 (P5726, Sigma) and 3 (P0044, Sigma). Protein concentrations were determined by the Pierce™ BCA protein assay (23225, Thermo Fisher Scientific, Waltham, MA, United States), and Bis-Tris SDS PAGE gels (3450124, Bio-rad, Hercules, CA, United States) were loaded with 30 μg protein, denatured by adding Dithiothreitol (DTT, 0.1M) and boiling at 95°C. The gel was transferred using a turbo transfer system (1704150, Bio-Rad) on a nitrocellulose membrane (1704159, Bio-rad). Blots were probed with primary antibodies anti-CAMK2G (HPA040656, Sigma, 1:1000), anti-ACTIN (MAB1501R, Sigma, St Louis, MO, United States, 1:20 000), anti-CAMK2A [NB100-1983 (clone 6G9), Novus Biologicals, Abingdon, United Kingdom, 1:20 000], and anti-CAMK2B (13-9800, Invitrogen, Waltham, MA, United States, 1:10 000) for 2 h at room temperature or overnight at 4°C. Blots were probed with IRDye^®^ secondary antibodies (800CW Goat anti-Mouse, 680LT Goat anti-Rabbit, LI-COR, Lincoln, NE, United States, 1:15 000) for 1 h at room temperature. Blots were developed on an Odyssey^®^ Imager (LI-COR) and quantified with Image Studio™ Lite software (LI-COR).

### RNA-seq analysis

To look at the distribution of *Camk2g* splice variants, we utilized an RNA-seq dataset that measured the transcriptome of the forebrain and cerebellum in adult male C57BL/6 mice (GEO: GSE141252; PRJNA592965; Stoeger et al., 2019). We imported raw sequencing data of the two brain regions of 4 mice (mouse number 10, 12, 13, and 25) to the Galaxy web platform (Afgan et al., 2018) and assessed the quality of the reads by FastQC (Galaxy Version 0.73 + galaxy0).^1^ Single-end reads were mapped to the mouse reference genome (mm39 Full) using the RNA star algorithm (Galaxy Version 2.7.8a; Dobin et al., 2013). Generated.bam files were opened in Integrative Genomics Viewer (version 2.11.9), and Sashimi plots of the *Camk2g* transcript were analyzed for the number of junctions between exons. Sashimi plots of the *Camk2g* linker domain are shown in Supplementary Figure 3. The number of splice junctions was averaged across the 4 mice and compared between the brain regions. To calculate the ratio of splicing, the number of times the exon was spliced out was divided by the average number of times the exon was spliced in; for example, for exon 13, the number of splice junctions from exon 12–14 (exon 13 spliced out) was divided by the average number of splice junctions from exon 12–13 and exon 13–14 (exon 13 spliced in).

### Behavior

Mice were handled by an experimenter blinded to their genotype before the start of experiments. The order of experiments was as follows: rotarod, Morris water maze, and fear conditioning. In a separate cohort: rotarod, open field, marble burying, grip strength, nest building, forced swim, and balance beam test.

#### Rotarod

Mice were placed on an accelerating cylinder (4–40 rpm; Ugo Basile Biological Research Apparatus, model 7650) for 5 consecutive days with 2 trials per day. A trial lasted for a maximum of 5 min, and the daily trials had a 45–60 min interval. The experimenter manually scored how long a mouse would stay on the rotarod or how long it clung to the cylinder for 3 consecutive rotations without walking. Sample sizes were 19 female and 26 male mice for *Camk2g*^+/+^ (*n* = 45), and 22 female and 23 male mice for *Camk2g*^–/–^ (*n* = 45).

#### Morris water maze

Mice were trained in a circular pool of 1.2 m to find a round platform of 11 cm that was submerged (1 cm) in cloudy water at 25–26°C. The training lasted 5 consecutive days with two trials per day in which the mouse was released at a pseudorandom location and had 60 s to locate the platform. The latency to find the platform was scored manually, and if the mouse did not find the platform within 60 s, it was guided by the experimenter. In between trials, the mouse rested on the platform for 30 s. The probe trial started with placing the mouse on the platform for 30 s, after which the platform was removed and the mouse was tracked using EthoVision^®^ software (Noldus^®^, Wageningen, Netherlands). The probe trial was executed on day 5. Sample sizes were 6 female and 9 male mice for *Camk2g*^+/+^ (*n* = 15), and 9 female and 6 male mice for *Camk2g*^–/–^ (*n* = 15).

#### Fear conditioning

Mice were placed in a soundproof box (26 cm × 22 cm × 18 cm; San Diego Instruments, San Diego, CA, United States) with a grid floor, white light turned on, and a camera monitoring motor activity. Conditioning consisted of placing the mice in a lightened soundproof box (26 × 22 × 18 cm; San Diego Instruments) and after 150 s presenting them with 85 dB tone for 20 s, followed by a single foot shock of 1.0 mA that lasted 2 s. Contextual memory was tested 24 h later by placing the mice in the same context for 180 s and measuring the amount of freezing by the camera. Again, 24 h later, cued memory was tested by placing the mice in a modified context for 220 s. The modification consisted of making the box dark, placing plexiglass walls in a triangular shape, removing the grid floor, and presenting them with a mild acetone smell. After 120 s, mice were presented with the conditioned stimulus, i.e., the tone, for 100 s, and the amount of freezing was measured by the camera. Freezing behavior was defined as no activity for 1.00 s and analyzed by Video Freeze^®^ software (Med Associates Inc., Fairfax, VT, United States). Sample sizes were 6 female and 9 male mice for *Camk2g*^+/+^ (*n* = 15), and 9 female and 6 male mice for *Camk2g*^–/–^ (*n* = 15). A separate cohort was tested for only contextual fear conditioning that consisted of 7 female and 8 male mice for *Camk2g*^+/+^ (*n* = 15), and 6 female and 9 male mice for *Camk2g*^–/–^ (*n* = 15).

#### Open field

Mice were placed facing the wall in a brightly lit circular open arena of 110 cm in diameter. Their activity was tracked for 10 min using EthoVision^®^ software (Noldus^®^). Sample sizes were 6 female and 9 male mice for *Camk2g*^+/+^ (*n* = 15), and 7 female and 8 male mice for *Camk2g*^–/–^ (*n* = 15).

#### Grip strength

Mice were placed on the grid of the grip strength test (Bioseb, Vitrolles, France) that was set at an angle of 30°. The grip strength of the fore and hind limbs was simultaneously measured by steadily pulling the tail in a horizontal direction. This was repeated 3 times per mouse. Sample sizes were 6 female and 9 male mice for *Camk2g*^+/+^ (*n* = 15), and 7 female and 8 male mice for *Camk2g*^–/–^ (*n* = 15).

#### Balance beam

Mice were placed on a platform and motivated with the gentle guidance of the hand to cross a beam 1 m long to a second platform that was closed. Beams were 12, 10, or 8 mm wide and were suspended at 50 cm in the air. Mice crossed the beams 3 times on 3 consecutive days; on day 1, mice were tested on beams of 12 and 10 mm, on day 2 mice were tested on beams of 10 and 8 mm, and on day 3 mice were tested on beams of 12, 10, and 8 mm. Videos were recorded and manually analyzed offline for the number of foot slips and latency. The figure reports the results when crossing the specific beam for the second day. Sample sizes were 5 female and 4 male mice for *Camk2g*^+/+^ (*n* = 9), and 4 female and 5 male mice for *Camk2g*^–/–^ (*n* = 9).

#### Marble burying

Mice were placed in an open Makrolon cage (50 cm × 26 cm × 18 cm) containing a 4-cm bedding (Lignocel Hygenic Animal Bedding, JRS) with 20 blue glass marbles equally distributed on top. After 30 min, mice were gently removed from the cages, and the number of marbles buried (>50% covered) was scored manually. Sample sizes were 6 female and 9 male mice for *Camk2g*^+/+^ (*n* = 15), and 7 female and 8 male mice for *Camk2g*^–/–^ (*n* = 15).

#### Nest building

For this experiment, mice were single-caged 5–7 days beforehand. The former nest building material was replaced by extra thick blot filter paper (11 g; 1703969, Bio-rad). Paper that was not used to build a nest was weighed every 24 h for 5 consecutive days. Sample sizes were 6 female and 9 male mice for *Camk2g*^+/+^ (*n* = 15), and 7 female and 8 male mice for *Camk2g*^–/–^ (*n* = 15).

#### Forced swim test

Mice were placed in a glass cylinder (diameter 18 cm and height 27 cm) that was filled with water at 26 ± 1°C up to 15 cm. Trials lasted 6 min, of which the first 2 min were for the mice to get habituated to the situation and the final 4 min were scored manually for immobility, defined as no activity other than that needed for the mice to remain afloat and keep its balance. Sample sizes were 6 female and 9 male mice for *Camk2g*^+/+^ (*n* = 15), and 7 female and 8 male mice for *Camk2g*^–/–^ (*n* = 15).

### Electrophysiology

Mice were sedated by inhalation of isoflurane and decapitated. The brains were quickly removed and placed in a vibratome (PELCO easiSlicer™, Ted Pella, Inc., Redding, CA, United States) with ice-cold artificial cerebrospinal fluid (ACSF; in mM: 120 NaCl, 3.5 KCl, 2.5 CaCl2, 1.3 MgSO4, 1.25 NaH2PO4, 26 NaHCO3, and 10 D-glucose). Sagittal slices of 400 μm were made from which the hippocampus was isolated and left to recover for 60 min in oxygenated (95%) and carbonated (5%) ACSF at room temperature. Bipolar platinum/iridium stimulating and recording electrodes (FHC; Bowdoin, ME, United States) were placed in the CA3-CA1 Schafer collateral pathway and the slices were left to habituate for 30 min. The input/output paradigm was executed by stimulation of 10, 20, 40, 60, 80, and 100 mA at 0.05 Hz. Paired-pulse facilitation was stimulated at one-third of max field excitatory postsynaptic potential (fEPSP) as assessed by the input/output. Paired pulses were given with an interval of 10, 25, 50, 100, 200, and 400 μs at 0.05 Hz, and this was repeated two times. LTP recordings were at 1 Hz and consisted of a baseline recording of 10 min, followed by LTP induction and subsequent recordings for 60–120 min. Induction of 100 Hz lasted for 1 s and stimulation was done at one-third max fEPSP. Theta induction was done at two-thirds max fEPSP strength with a burst of 4 stimuli at 100 Hz, repeated 10, 3, or 2 times, with an interval of 200 μs between stimulation bursts. Recordings took place in submerged chambers with a flow of 2 ml/min of ACSF at 30°C. Analysis was done in pCLAMP 11 software (Molecular Devices, San Jose, CA, United States), measuring the slope of the fEPSP and normalizing it to the baseline. Recordings that showed an unstable response during baseline were excluded.

### Statistical analysis

Statistical analysis was done with GraphPad Prism software. Behavioral data were tested for normality with the D’Agostino-Pearson test. Subsequently, parametric or non-parametric tests were used, and *P*-values of <0.05 were considered significant. Details about the statistical tests used and their exact values are indicated in the figure legends together with the sample sizes.

## Results

**FIGURE 1:**
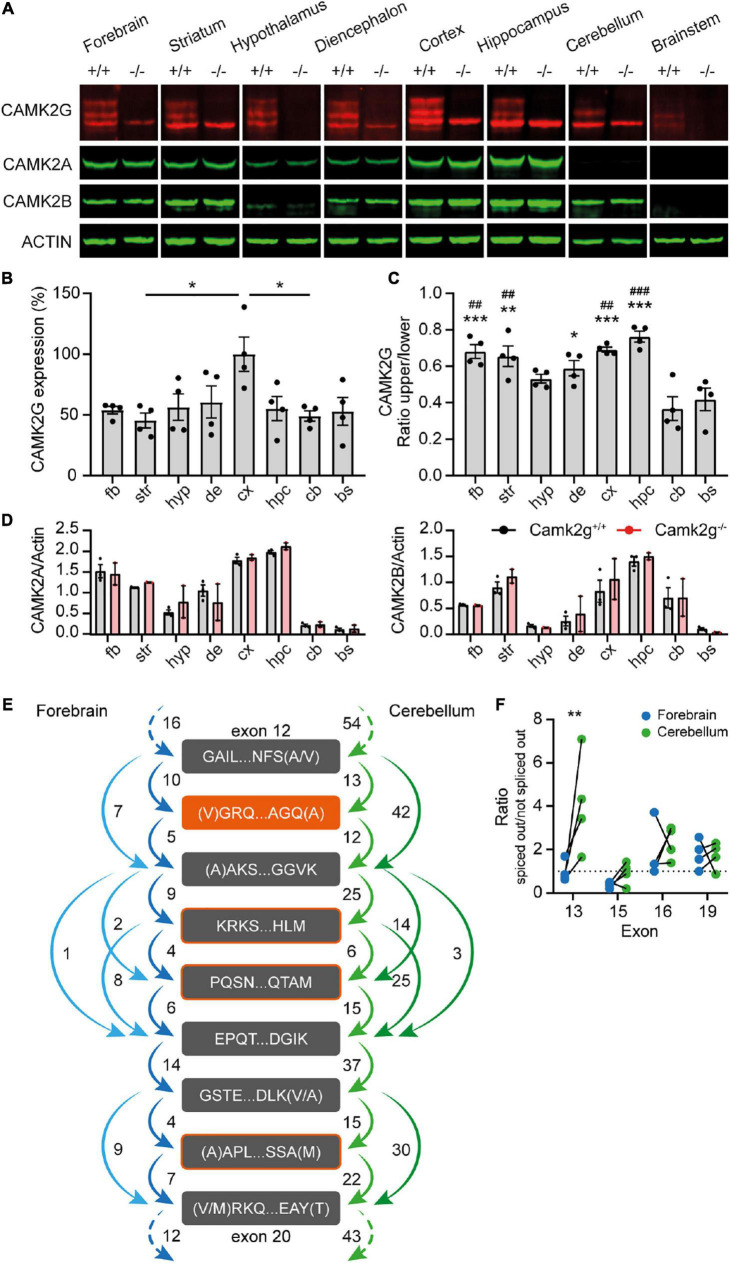
CAMK2G expression profile in the brain. **(A)** Immunoblots of different isolated brain regions in *Camk2g* wild type (+/+) and knockout (−/−) mice probed with antibodies against CAMK2G, CAMK2A, CAMK2B, and ACTIN. Note the triple band of CAMK2G, of which 2 are absent in *Camk2g*^−/−^ mice. **(B)** Quantification of CAMK2G expression, normalized to values in the cortex. fb, forebrain; str, striatum; hyp, hypothalamus; de, diencephalon; cx, cortex; hpc, hippocampus; cb, cerebellum; bs, brainstem [one-way ANOVA with Bonferroni’s *post hoc* analysis, *F*_(7_,_24)_ = 2.98, *p* = 0.021; fb vs. str *p* > 0.999, fb vs. hyp *p* > 0.999, fb vs. bg *p* > 0.999, fb vs. cx *p* = 0.088, fb vs. hpc *p* > 0.999, fb vs. cb *p* > 0.999, fb vs. bs *p* > 0.999, str vs. hyp *p* > 0.999, str vs. bg *p* > 0.999, str vs. cx *p* = 0.019, str vs. hpc *p* > 0.999, str vs. cb *p* > 0.999, str vs. bs *p* > 0.999, hyp vs. bg *p* > 0.999, hyp vs. cx *p* = 0.134, hyp vs. hpc *p* > 0.999, hyp vs. cb *p* > 0.999, hyp vs. bs *p* > 0.999, bg vs. cx *p* = 0.272, bg vs. hpc *p* > 0.999, bg vs. cb *p* > 0.999, bg vs. bs *p* > 0.999, cx vs. hpc *p* = 0.109, cx vs. cb *p* = 0.037, cx vs. bs *p* = 0.073, hpc vs. cb *p* > 0.999, hpc vs. bs *p* > 0.999, cb vs. bs *p* > 0.999; *n* = 4]. **(C)** Ratio of the specific CAMK2G bands. The lower band shows higher expression levels throughout the brain, but exceptionally so in cerebellum and brainstem, *indicates significance compared to cerebellum and # compared to brainstem [one-way ANOVA with Bonferroni’s post-hoc analysis compared to Cb and Bs, *F*_(7,24)_ = 9.57, *p* < 0.001; fb vs. cb *p* < 0.001, fb vs. bs *p* = 0.003, str vs. cb *p* = 0.001, str vs. bs *p* = 0.008, hyp vs. cb *p* = 0.115, hyp vs. bs *p* = 0.615, de vs. cb *p* = 0.013, de vs. bs *p* = 0.090, cx vs. cb *p* < 0.001, cx vs. bs *p* = 0.002, hpc vs. cb *p* < 0.001, hpc vs. bs *p* < 0.001, and cb vs. bs *p* > 0.999]. **(D)** CAMK2A (left) and CAMK2B (right) expression differs between brain regions, but importantly does not differ between *Camk2g*^+/+^ and *Camk2g^−/−^* [two-way Anova, CAMK2A *F*_(7_,_24)_ = 0.245, *p* = 0.625; CAMK2B *F*_(7_,_24)_ = 0.809, *p* = 0.378; *n* = 2–3]. **(E)** Average amount of reads between exons of *Camk2g* in RNA-seq database on the forebrain (blue) and the cerebellum (green). The start and end of the amino acid sequence are indicated for the exons. Splice variants only occur on exons 13 (orange), 15, 16, and 19 (outlined in orange). **(F)** Ratio of when the exon is spliced out versus not spliced out in the forebrain and the cerebellum, lines indicate quantifications within mice. Only exon 13 shows a significant ratio difference between the forebrain and the cerebellum, with exon 13 more often spliced out in the cerebellum [two-way ANOVA with Bonferroni’s *post hoc* analysis on exon splicing, interaction: *F*_(3_,_24)_ = 3.86, *p* = 0.022; exon 13 *p* = 0.001, exon 15, 16, and 19 *p* > 0.999; *n* = 4]. Data represents mean ± SEM; *, #*p* < 0.05, **, ##*p* < 0.01, and ***, ###*p* < 0.001.

*Camk2g* knockout mice (EUCOMM; *Camk2g*^–/–^) were bred in a C57BL/6J background. For all experiments, wild-type littermates (*Camk2g*^+/+^) were used as control. *Camk2g^–/–^* were viable and appeared healthy, but showed reduced body weight compared with *Camk2g*^+/+^ (Supplementary Figure 1). To validate the *Camk2g^–/–^* mice and assess where CAMK2G is expressed, we isolated 8 brain regions from *Camk2g^–/–^* and *Camk2g*^+/+^ mice and performed Western blots on forebrain, striatum, hypothalamus, diencephalon, cortex, hippocampus, cerebellum, and brainstem. Probing for CAMK2G revealed three bands, of which the upper two were specific and were absent in the *Camk2g^–/–^* samples (Figure 1A), suggesting different isoforms of CAMK2G are expressed in the brain. In general, CAMK2G is expressed quite consistently throughout the brain, with a trend for the highest expression levels in the cortex (Figures 1A, B; for statistics see the legends). As expected, CAMK2G is absent in all brain regions of the *Camk2g^–/–^* samples, while CAMK2A and CAMK2B expressions were unaltered compared to *Camk2g*^+/+^ (Figures 1A, D). The lowest aspecific band of CAMK2G overlapped with CAMK2B (for raw blots see Supplementary Figure 2). Interestingly, the two CAMK2G isoforms showed a region-defined expression: in regions from the cerebrum, both isoforms were nearly equally expressed, but in the cerebellum and brainstem, expression of the larger isoform was markedly decreased (Figures 1A, C).

To confirm the presence of different CAMK2G isoforms in different brain areas, an online RNA-seq database (GEO accession number: GSE141252; Stoeger et al., 2019 bioRxiv) on the forebrain and cerebellum tissue of adult C57BL/6J mice was used to quantify exon-junctions of the *Camk2g* transcripts. Confirming literature (Takeuchi et al., 2000; Tombes et al., 2003; Kamata et al., 2006; Sloutsky and Stratton, 2021), we found splice variants solely in the linker domain of *Camk2g*, splicing out exon 13, 15, 16, and/or 19 (for numbering of exons see Supplementary Figure 3). Figure 1E illustrates exons in the linker domain with the amount of transcript reads between exons (for raw data plots, see Supplementary Figure 4). Intriguingly, exon 13 showed the largest difference between brain regions and is spliced out more frequently in the cerebellum than in the forebrain (Figure 1F), which would translate to a 1.1 kDa decrease in size on protein level, corresponding to our Western blot results (Figure 1A). Therefore, the observed different ratio in cerebrum versus cerebellum protein expression is likely due to region-specific CAMK2G isoform distribution.

**FIGURE 2:**
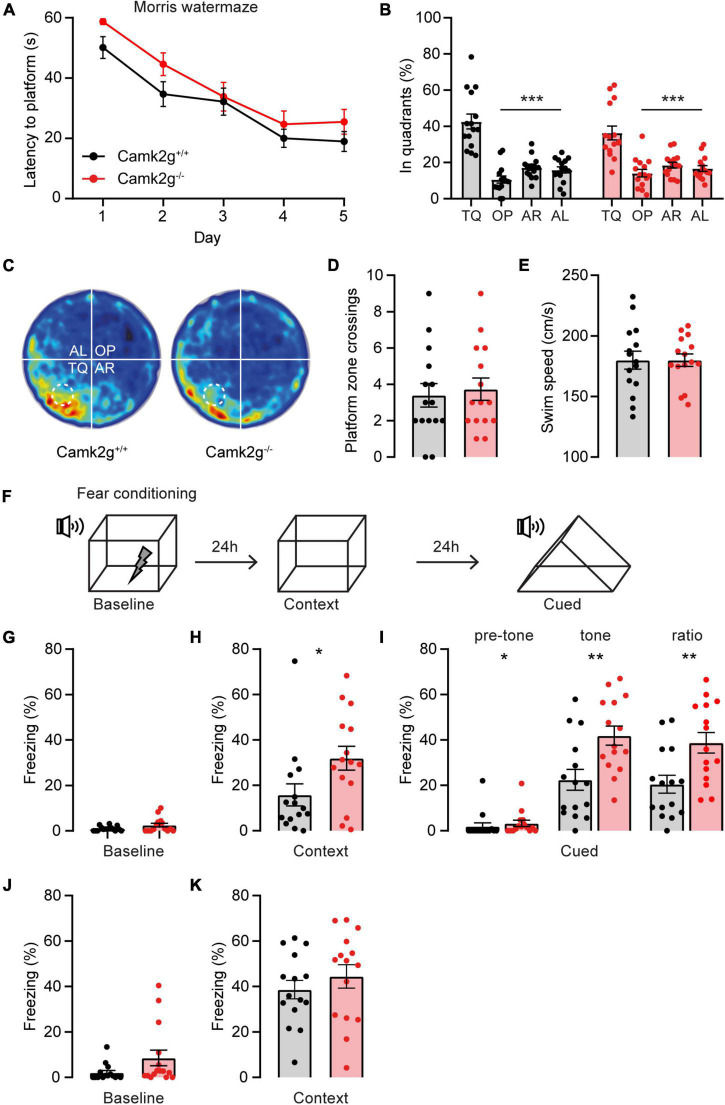
CAMK2G in cognitive behavior. **(A)** *Camk2g*^+/+^ and *Camk2g*^–/–^ mice similarly showed reduced latencies to find the platform over days [two-way repeated measures ANOVA; interaction: *F*_(4_,_112)_ = 0.41, *p* = 0.804]. **(B)** Both groups spent significantly more time in the target quadrant (TQ) compared with other quadrants in the probe trial on day 5 [one-way ANOVA with Bonferroni’s *post hoc* analysis. *Camk2*^+/+^: *F*_(3_,_56)_ = 31.61, *p* < 0.001, TQ vs. OP *p* < 0.001, TQ vs. AR *p* < 0.001, TQ vs. AL *p* < 0.001; *Camk2*^–/–^: itF_(3_,_56)_ = 17.04, *p* < 0.001, TQ vs. OP *p* < 0.001, TQ vs. AR *p* < 0.001, TQ vs. AL *p* < 0.001]. TQ, target quadrant; OP, opposite; AR, adjacent right; AL, adjacent left. **(C)** Heat map of the probe trial, the arena is divided into four quadrants and the location where the platform was located during learning is indicated with a dashed line. **(D)** During the probe trial, mice crossed the zone where the platform was situated during learning with a similar frequency [unpaired *t*-test, *t*_(28)_ = 0.37, *p* = 0.711]. **(E)** Swim speed velocity was similar between groups during the probe trial [unpaired *t*-test, *t*_(28)_ = 0.0018, *p* = 0.999]. **(F)** Schematic representation of the fear conditioning test. **(G)** Freezing behavior at baseline showed no significant difference between *Camk2g*^+/+^ and *Camk2g^–/–^* (Mann–Whitney *U* test, *U* = 84.5, *p* = 0.245). **(H)** When placed in the same context, *Camk2g^–/–^* showed significantly more freezing (Mann–Whitney *U* test, *U* = 60, *p* = 0.030). **(I)** When put in a different context, *Camk2g^–/–^* already showed more freezing than *Camk2g*^+/+^ pretone (Mann–Whitney *U* test, *U* = 64, *p* = 0.026). Upon exposure to the tone, *Camk2g^–/–^* again showed significantly more freezing than *Camk2g*^+/+^ [unpaired *t*-test, *t*_(28)_ = 3.13, *p* = 0.004]. The ratio was calculated by subtracting pre-tone from tone freezing levels and still showed significantly more freezing in *Camk2g^–/–^* than *Camk2g*^+/+^ [unpaired *t*-test, *t*_(28)_ = 3.02, *p* = 0.005]. **(J)** In a separate cohort, mice were tested exclusively for contextual fear conditioning and showed no differences in freezing behavior at baseline (Mann–Whitney *U* test, *U* = 71, *p* = 0.083). **(K)** When put in the same context 24 h later, *Camk2g*^–/–^showed no differences in freezing compared to *Camk2g*^+/+^ [unpaired *t*-test, *t*_(28)_ = 0.885, *p* = 0.384]. Data represents mean ± SEM (*n* = 15); * *p* < 0.05, ** *p* < 0.01, *** *p* < 0.001.

Having established that our mouse model is indeed lacking CAMK2G expression, we set out to study the role of CAMK2G in learning and plasticity. For spatial memory, we made use of the Morris water maze. Although the *Camk2g^–/–^* mice show slightly longer latencies to find the platform in the first 2 days compared with *Camk2g*^+^*^/^*^+^ mice, all mice showed a reduction in their latency to find the platform over days, with no differences between genotypes over days (Figure 2A). Reduction in latency does not necessarily mean that the mouse shows spatial learning as mice can use different non-hippocampus-dependent strategies to find the platform. Hence, to assess hippocampus-dependent spatial memory, the platform was removed for a probe trial on day 5 and the time spent in the different quadrants of the water maze was analyzed. Both *Camk2g*^+/+^ and *Camk2g^–/–^* spent significantly more time in the target quadrant (TQ) compared with the other quadrants (Figures 2B, C), indicating intact, or at best very moderately impaired, spatial learning in our *Camk2g^–/–^* mouse model. Additionally, no differences were found in the number of platform crosses (Figure 2D) or the swim speed (Figure 2E).

We next tested associative learning using contextual and cued fear conditioning. Mice were placed in a context, where after 150 s a tone was presented for 20 s, immediately followed by a foot shock (Figure 2F). No significant differences in freezing behavior were observed between the *Camk2g*^+/+^ and *Camk2g^–/–^* mice during baseline measurement (Figure 2G). Surprisingly, *Camk2g^–/–^* showed significantly more freezing than *Camk2g*^+/+^ in the context condition (Figure 2H). Also in the cued condition, *Camk2g^–/–^* showed significantly more freezing than *Camk2g*^+/+^; however, baseline freezing was already significantly different in the *Camk2g^–/–^* mice compared with their wild-type littermates. To assess the learning effect, pre-tone freezing levels were subtracted from tone freezing levels, which still revealed significantly higher freezing levels in *Camk2g^–/–^* than *Camk2g*^+/+^ (Figure 2I). Combining the tone and shock strongly activates the amygdala, potentially masking the hippocampus-dependent contribution of this conditioning task. To explicitly test the hippocampal contribution to the phenotype, in a separate cohort we only tested contextual conditioning, omitting the cue. Again, no significant difference was observed in baseline freezing levels (Figure 2J). When placed back in the same context 24 h later, similar freezing levels were observed between *Camk2g*^+/+^ and *Camk2g^–/–^* (Figure 2K). Thus, our results suggest that CAMK2G is not necessary for hippocampus-dependent learning and memory, but potentially plays a role in amygdala-dependent associative fear conditioning.

**FIGURE 3:**
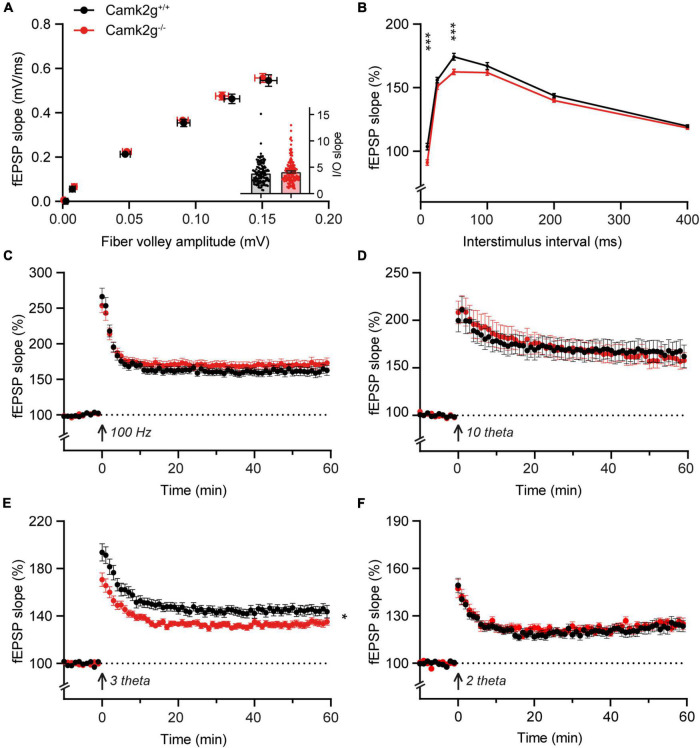
CAMK2G in synaptic plasticity. **(A)** Field recordings were done in the Schaffer-collateral-CA1 synapses of the hippocampus. At baseline synaptic transmission, mice did not show differences in the input/output (I/O) between the presynaptic fiber volley and postsynaptic fEPSP with increasing stimulus strength [unpaired *t*-test on slopes of curve (inset), *t*_(268)_ = 1.01, *p* = 0.314. Fiber volley *n* = 126/21 slices/mice for *Camk2g*^+/+^ and *n* = 144/24 for *Camk2g*^–/–^; fEPSP *n* = 144/21 for *Camk2g*^+/+^ and *n* = 162/24 for *Camk2g*^–/–^]. **(B)** We tested presynaptic short-term plasticity with paired-pulse facilitation and showed differences between *Camk2g*^+/+^ and *Camk2g^– /–^* mice at interstimulus intervals of 10 and 50 ms [unpaired *t*-tests, 10 ms: *t*_(197)_ = 3.70, *p* < 0.001; 25 ms: *t*_(197)_ = 1.65, *p* = 0.101; 50 ms: *t*_(197)_ = 3.45, *p* < 0.001; 100 ms: *t*_(197)_ = 1.54, *p* = 0.125; 200 ms: *t*_(197)_ = 1.82, *p* = 0.071; 400 ms: *t*_(197)_ = 0.94, *p* = 0.352; *n* = 95/15 for *Camk2g*^+/+^ and *n* = 104/17 for *Camk2g*^–/–^]. **(C)** No differences were found in LTP elicited by stimulation at 100-Hz for 1 s [two-way repeated measures ANOVA on final 10 min, genotype: *F*_(1_,_77)_ = 1.01, *p* = 0.318; *n* = 37/7 for *Camk2g*^+/+^ and *n* = 42/8 for *Camk2g*^–/–^]. **(D)** No differences were found in LTP elicited by 10-theta stimulation [two-way repeated measures ANOVA on final 10 min, genotype: *F*_(1_,_35)_ = 0.25, *p* = 0.622; *n* = 19/5 for *Camk2g*^+/+^ and *n* = 18/5 for *Camk2g*^–/–^]. **(E)** *Camk2g^–/–^* mice showed reduced post-tetanic potentiation and LTP elicited by 3-theta stimulation [two-way repeated measures ANOVA on final 10 min, genotype: *F*_(1_,_80)_ = 7.02, *p* = 0.010; *n* = 41/11 for *Camk2g*^+/+^ and *n* = 42/12 for *Camk2g*^–/–^]. **(F)** No differences were found in LTP elicited by 2-theta stimulation [two-way repeated measures ANOVA on final 10 min, genotype: *F*_(1_,_59)_ = 0.12, *p* = 0.734; *n* = 28/9 for *Camk2g*^+/+^ and *n* = 33/10 for *Camk2g*^–/–^]. Data represents mean ± SEM; * *p* < 0.05, *** *p* < 0.001.

Having assessed hippocampal learning, we set out to investigate hippocampal synaptic plasticity in *Camk2g^–/–^*, which could potentially still reveal a mild phenotype. We performed extracellular field recordings in the CA1 to CA3 Shaffer collateral pathway and detected no differences in baseline synaptic transmission since the presynaptic fiber volley and the field excitatory postsynaptic potential (fEPSP) increased equally between *Camk2g*^+/+^ and *Camk2g^–/–^* mice with increasing stimulus strength (Figure 3A). We detected a significant decrease in the paired-pulse facilitation in *Camk2g^–/–^* compared with *Camk2g*^+/+^, but this was only apparent at the 10- and 50-ms intervals (Figure 3B). Induction of long-term potentiation (LTP) using a 100-Hz induction protocol resulted in LTP in both *Camk2g*^+/+^ and *Camk2g*^–/–^, with no significant difference neither after 60 min nor after 120 min recording between the two groups (Figure 3C and Supplementary Figure 5). As the classical 100-Hz stimulation is very strong, it is possible that a plasticity phenotype is masked, hence, milder LTP induction protocols were tested. No differences in LTP were found between Camk2g^+/+^ and Camk2g^–/–^ in the 10-theta condition; however, Camk2g^–/–^ showed significantly impaired LTP compared to Camk2g^+/+^ in the 3-theta induction condition (Figures 3D, E). Surprisingly, this difference was lost in the 2-theta condition (Figure 3F). These results suggest that if there is a role for CAMK2G in synaptic plasticity at the CA3-CA1 synapse, it is very mild.

**FIGURE 4:**
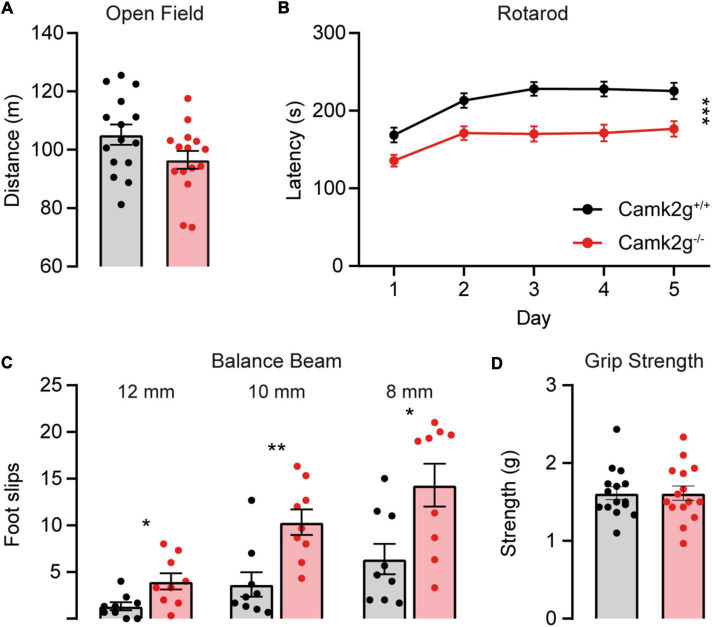
CAMK2G in motor behavior. **(A)** No differences were found in the distance traveled in the open field [unpaired *t*-test, *t*_(28)_ = 1.85, *p* = 0.076; *n* = 15]. **(B)** *Camk2g^–/–^* mice showed reduced latency to fall of the accelerating rotarod [two-way repeated measures ANOVA; interaction: *F*_(4_,_352)_ = 1.51, *p* = 0.200; genotype: *F*_(1_,_88)_ = 18.40, *p* < 0.001; *n* = 45]. **(C)** *Camk2g^–/–^* mice consistently slipped more often when crossing beams that were 12, 10, and 8 mm wide [unpaired *t*-test or Mann–Whitney *U* test; 12 mm: *t*_(28)_ = 2.75, *p* = 0.014; 10 mm: *U* = 8.5, *p* = 0.003; 8 mm: *t*_(28)_ = 2.81, *p* = 0.013; *n* = 9]. **(D)** Grip strength was unaltered between *Camk2g*^+/+^ and *Camk2g^–/–^* mice [Mann–Whitney *U* test, *U* = 111, *p* = 0.959; *n* = 15]. Data represents mean ± SEM; * *p* < 0.05, ** *p* < 0.01, *** *p* < 0.001.

**FIGURE 5:**
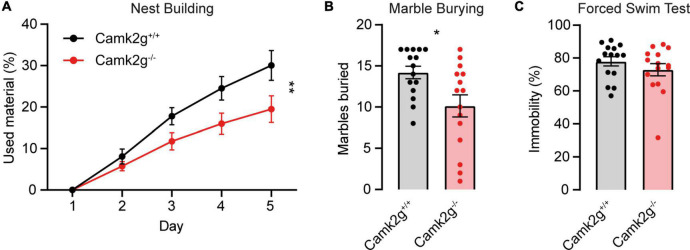
CAMK2G in intrinsic behavior. **(A)** *Camk2g^–/–^* mice used less material to build nests than *Camk2g*^+/+^ mice [two-way repeated measures ANOVA; interaction: *F*_(4_,_112)_ = 4.55, *p* = 0.002]. **(B)** *Camk2g^–/–^* mice buried less marbles than *Camk2g*^+/+^ mice [unpaired *t*-test, *t*_(28)_ = 2.65, *p* = 0.013]. **(C)** No differences were found in the amount of time the mice spent immobile in the forced swim test (Mann–Whitney *U* test, *U* = 90.5, *p* = 0.372). Data represent mean ± SEM (*n* = 15); * *p* < 0.05, ** *p* < 0.01.

To further study the effect of the absence of CAMK2G on behavior and brain function, we expanded our tests to include other forms of behavior such as motor and innate behavior. We tested locomotor activity in the open field and found a trend toward decreased activity in *Camk2g^–/–^* compared with *Camk2g*^+/+^, though not significant (Figure 4A). We further challenged their motor performance as well as their motor learning using the accelerating rotarod. Although both the *Camk2g^–/–^* and *Camk2g*^+/+^ mice showed motor learning over days, the *Camk2g^–/–^* performed significantly poorer at all time points measured (Figure 4B). Also, on the balance beam, sensitive to balance and fine motor coordination, *Camk2g^–/–^* consistently showed more foot slips crossing beams of varying widths (Figure 4C). Since *CAMK2G* patients show developmental hypotonia (Proietti Onori et al., 2018), we tested if reduced muscle strength could play a role in the reduced motor performance seen in *Camk2g^–/–^* mice, but this was not the case as grip strength was equal between the groups (Figure 4D). Finally, we assessed some innate behaviors of the *Camk2g*^–/–^ using the nest building, marble burying, and forced swim test. Compared with *Camk2g*^+/+^, *Camk2g^–/–^* used significantly less material to build a nest over days (Figure 5A) and showed impaired burying behavior (Figure 5B). No significant differences were observed between *Camk2g^–/–^* and *Camk2g*^+/+^ mice in floating behavior in the forced swim test (Figure 5C).

## Discussion

With the identification of patients suffering from *CAMK2G-*associated neurodevelopmental disorder (de Ligt et al., 2012; Proietti Onori et al., 2018), the interest to study the role of CAMK2G in normal brain functioning significantly increased. Here, we set out to expand the knowledge on the role of CAMK2G in neuronal functioning through the characterization of a novel *Camk2g^–/–^* mouse model. We provide evidence for differential expression of different CAMK2G isoforms depending on the brain area, where we speculate that this is due to differential splicing of exon 13. At the behavioral level, our results suggest that CAMK2G has a minor role in hippocampus-dependent spatial learning and memory, as well as synaptic plasticity, but does affect amygdala-dependent fear learning. Finally, we find clear phenotypes in fine motor performance and intrinsic behaviors, as measured using the nest-building and marble-burying assays.

The expression levels of CAMK2G throughout the brain were assessed by isolating different brain regions of *Camk2g*^+^*^/^*^+^ and *Camk2g^–/–^* mice. Isolation of brain regions was validated by CAMK2A expression, which was highest in the hippocampus and the cortex and lowest in the cerebellum and the brainstem, and CAMK2B expression, also highest in the hippocampus and dominant in the cerebellum yet nearly absent in the brainstem (Cook et al., 2018). Total levels of CAMK2G did not differ much between the different brain areas. Interestingly, in brain areas where CAMK2B and CAMK2A showed little expression, such as the hypothalamus, CAMK2G appeared to be one of the more dominating CAMK2 isozymes. However, we have to be careful in drawing such conclusion as we did not use a pan-CAMK2 antibody to directly compare expression levels between CAMK2s. Even though the CAMK2G antibody also recognized CAMK2B (see raw blots in Supplementary Figure 2), which runs at the height of the lowest band, it is interesting to see that in the hypothalamus and the brainstem, the lowest band can still be seen in the WT but no longer in the knockout samples. As CAMK2B is hardly detectable in these brain areas and this lowest band disappears in *Camk2g^–/–^* samples, these findings could suggest the expression of a third smaller isoform of CAMK2G. A more specific antibody that does not cross-react with CAMK2B would be necessary to substantiate this conclusion. For the upper two CAMK2G bands, we find evidence for differential expression of different CAMK2G isoforms. Based on RNA-seq data, the different isoforms correspond with differential splicing of exon 13. Our data suggest that the isoform containing this exon is expressed in the cerebrum but hardly in the cerebellum. Interestingly, the most common transcript found in the human hippocampus does not contain this exon as the number of transcripts with exon 13 detected was <1% (Sloutsky et al., 2020). Whether CAMK2G in the human brain shows region-specific isoforms or whether this differential expression of isoforms is species-specific remains to be studied. A homolog of exon 13 is expressed by CAMK2B, sharing 55% similarity with exon 13 in CAMK2G on the amino acid level, and encompasses the binding region for F-Actin (O’leary et al., 2006). Future research will have to show whether this exon in CAMK2G also mediates binding to F-Actin.

Our conclusion that CAMK2G has a minor role in hippocampal learning, memory, and cognition is in contrast with previous findings. The first study on a global *Camk2g^–/–^* mouse model showed impaired long-term (24 h) memory, as well as reduced late-phase LTP in *Camk2g^–/–^* (Cohen et al., 2018). Furthermore, they show that the deletion of CAMK2G solely in forebrain excitatory neurons is sufficient to cause the deficits seen in the global *Camk2g^–/–^* mice (Cohen et al., 2018). Interestingly, the following study showed that CAMK2G is mainly expressed in interneurons and that the deletion of CAMK2G specifically in parvalbumin-positive interneurons is sufficient to induce deficits in synaptic plasticity in these interneurons as well as in behavioral long-term (24 h) memory (He et al., 2021). It is challenging to explain the differences in findings between the different labs. One of the possible explanations is the differences in mouse strains used. The mice we used for our experiments were kept in the C57BL/6J background, whereas the original mice used in the other studies were in a 29SvEv/C57BL6/CD1 mixed background (Backs et al., 2010; Cohen et al., 2018; He et al., 2021). Indeed, it has been shown for other mouse models that mouse strain can make a significant difference in behavioral phenotypes (Born et al., 2017; Sonzogni et al., 2018). However, to test whether this is indeed the case for CAMK2G, the different mouse strains would need to be assessed side by side.

It might not be surprising that CAMK2G only has a minor role in hippocampus-dependent learning and plasticity. CAMK2A and CAMK2B, the two most abundant CAMK2 isozymes expressed in the brain, have both been shown to be essential for hippocampal plasticity and learning (Silva et al., 1992a,b; Elgersma et al., 2002; Borgesius et al., 2011), and the deletion of *Camk2a* and *Camk2b* simultaneously results in the complete absence of NMDA-receptor dependent LTP (Kool et al., 2019). If CAMK2G were to play a role in these processes as well, it should (partially) compensate for the lack of CAMK2A and CAMK2B, which it does not. Especially considering that CAMK2G is one of the earliest CAMK2 isozymes to be expressed during development; it can be detected already as early as E11.5, whereas CAMK2B starts to be expressed at E14.5 and CAMK2A around P1 (Bayer et al., 1999).

On the other hand, it is possible that CAMK2G affects different intracellular pathways than CAMK2A and/or CAMK2B, having a unique role in plasticity. For example, CAMK2G plays a role in the excitation–transcription coupling in excitatory neurons, shuttling calmodulin to the nucleus upon excitation (Ma et al., 2014; Cohen et al., 2016). This specific excitation–transcription shuttling could explain why the previous study in *Camk2g^–/–^* mice only found a difference in late-phase LTP and not in the early phase (Cohen et al., 2018). However, when testing late-LTP in our mouse model, we did not find a deficit (Supplementary Figure 5). The discrepancy in findings could be due to different induction protocols. We used a 1 × 100 Hz for 1 s induction protocol, whereas Cohen et al. (2018) used a stronger induction protocol: 3 × 100 Hz for 1 s with 5 min interstimulus interval. Whether these differences in induction protocols could result in differences in excitation–transcription coupling remains to be studied. The difference in mouse strains used is likely a better explanation for the discrepancy in findings between labs. Indeed, strain differences have been shown both for hippocampal learning and LTP (Nguyen et al., 2000). Finally, CAMK2G is not the only CAMK2 isozyme containing a nuclear localization signal (NLS), as this has also been shown for a CAMK2A isoform found in the midbrain/diencephalon regions (Brocke et al., 1995), and for CAMK2D (Srinivasan et al., 1994; Shioda et al., 2015). However, the exact role of the nuclear translocation of these isozymes remains to be elucidated.

There are several piles of evidence that suggest a role for CAMK2G in neurodevelopment: (1) the expression onset of *CAMK2G* is already very early in neurodevelopment, and at that developmental timepoint, CAMK2G and CAMK2D are the most abundant CAMK2 isozymes expressed in the brain (Proietti Onori et al., 2018); (2) CAMK2G is necessary for proper neurite formation during neuronal development *in vitro* (Proietti Onori et al., 2018; Xi et al., 2019), and (3) a pathogenic mutation in CAMK2G has been shown to cause a neurodevelopmental disorder in children (de Ligt et al., 2012; Proietti Onori et al., 2018). Our findings that CAMK2G plays an important role in motor and innate behavior further support the neurodevelopmental role for CAMK2G. Indeed, the behavioral tests assessed here, especially marble burying and rotarod, have been shown by us and others to have critical developmental periods, for example, in *Ube3a* and *Shank3* knockout mice (Silva-Santos et al., 2015; Mei et al., 2016; Rotaru et al., 2020; Sonzogni et al., 2020). Not only does adult *Ube3a* or *Shank3* gene reinstatement fail to rescue the marble burying or rotarod phenotypes but adult deletion of Ube3a also fails to induce phenotypes in these behavioral assays (Sonzogni et al., 2019). Another evidence for a neurodevelopmental origin for motor behavior comes from the inducible *Camk2b* knockout mouse. Deletion of *Camk2b* from the germline causes severe impairment in rotarod performance, whereas in mice where deletion of *Camk2b* is induced in adulthood, this phenotype is much milder (Kool et al., 2016). This is in stark contrast to cognitive behavior as, for example, tested in a conditional *Camk2a* mouse model. Deletion of *Camk2a* in adult mice is as detrimental for hippocampal learning and plasticity as germline deletion (Achterberg et al., 2014), and adult reinstatement rescues all cognitive behavior (Rigter et al., 2022). One important aspect here is the onset of expression. Whereas CAMK2G, CAMK2B, and UBE3A already have their expression onset prenatally, CAMK2A starts to be expressed postnatally; hence, early neurodevelopment is unaltered (Bayer et al., 1999; Judson et al., 2014).

Overall, our results reinforce previous literature suggesting that CAMK2G plays a critical role in normal brain functioning, albeit not in hippocampus-dependent memory, and further support the role of CAMK2G in neurodevelopment.

## Data availability statement

The original contributions presented in this study are included in the article/Supplementary material, further inquiries can be directed to the corresponding author.

## Ethics statement

The animal study was reviewed and approved in accordance with the European Commission Council Directive 2010/63/EU (CCD project license AVD101002017893), by an Independent Review Board (IRB) of the Erasmus MC.

## Author contributions

PR performed the experiments, analyzed the data, and wrote the manuscript. CK performed the experiments and was responsible for the mouse breedings and genotyping. GW designed the study and wrote the manuscript. All authors contributed to the article and approved the submitted version.

## Footnotes

1 Babraham Bioinformatics. *FastQC. A quality control tool for high throughput sequence data*. Available online at: https://www.bioinformatics.babraham.ac.uk/projects/fastqc/.
