# Supplementary material for: Loss of CAMK2G affects intrinsic and motor behavior but has minimal impact on cognitive behavior
Source: Front Neurosci. 2023 Jan 6;16:1086994. doi: 10.3389/fnins.2022.1086994 (PMC9853378; doi:10.3389/fnins.2022.1086994)
Supplement: Supplementary file 1 [file Image_1.PDF]

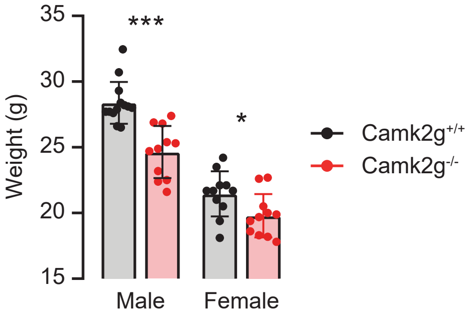

Supplementary Figure 1: *Camk2g*$^{-/-}$ have reduced body weight, here shown at 12 weeks old for both sexes (unpaired t-test, male $t(22) = 5.13$, $p < 0.001$; female $t(20) = 2.32$, $p = 0.031$; $n = 11\text{-}13$).
