# Supplementary material for: Loss of CAMK2G affects intrinsic and motor behavior but has minimal impact on cognitive behavior
Source: Front Neurosci. 2023 Jan 6;16:1086994. doi: 10.3389/fnins.2022.1086994 (PMC9853378; doi:10.3389/fnins.2022.1086994)
Supplement: Supplementary file 2 [file Image_2.PDF]

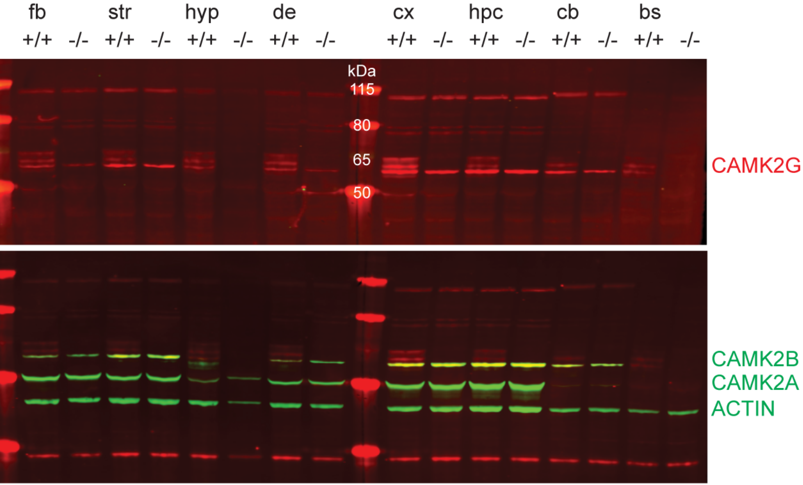

Supplementary figure 2. Raw immunoblots on different brain regions of Camk2g+/+ and Camk2g-/- mice probed with antibodies for CAMK2G (up), followed by CAMK2B, CAMK2A and ACTIN (down). fb = forebrain, str = striatum, hyp = hypothalamus, de = diencephalon, cx = cortex, hpc = hippocampus, cb = cerebellum, bs = brainstem.
