# Supplementary material for: Loss of CAMK2G affects intrinsic and motor behavior but has minimal impact on cognitive behavior
Source: Front Neurosci. 2023 Jan 6;16:1086994. doi: 10.3389/fnins.2022.1086994 (PMC9853378; doi:10.3389/fnins.2022.1086994)
Supplement: Supplementary file 3 [file Image_3.PDF]

Exon	Sequence (aa)
1	MATTATCTRFTDDYQLFEELG(K)
2	(K)GAFSVVRRCVKKTSTQEYAAKIINTKKLSAR(D)
3	(D)HQKLEREARICRLLKHPNI(V)
4	(V)RLHDSISEEGFHYLVFD(L)
5	(L)VTGGELFEDIVAREYYSEADA(S)
6	(S)HCIHQILESVNHIHQHDIVHRDLK
7	PENLLLASKCKGAAVKLADFGLAIEVQGEQQAWF(G)
8	(G)FAGTPGYLSPEVLRKDPYGKPVDIWAC(G)
9	(G)VILYILLVGYPPFWDEDQHKLYQQIKAGAYD
10	FPSPEWDTVTPEAKNLINQMLTINPAKRITADQALKHPWVC
11	QRSTVASMMHRQETVECLRKFNARRKLK
12	GAILTTMLVSRNFS(V/A)
13	(V)GRQSSAPASPAASAAGLAGQ(A)
14	(A)AKSLLNKKSDGGVK
15	KRKSSSSVHLM
16	PQSNNKNSLVSPAQEPAPLQTAM
17	EPQTTVVHNATDGIK
18	GSTESCNTTTEDEDLK(A/V)
19	(A)APLRTGNGSLVPEGRSPRDRTAPSAGMQPQPSLCSSA(M)
20	(M/V)RKQEIIKITEQLIEAINNGDFEAY(T)
21	(T)KICDPGLTSFEPEALGNLVEGMDFHKFYFEN(L)
22	(L)LSKNSKPIHTTILNPHVHVIGEDAACIAYIRLTQYIDGQGRPRTSQSEETRVWHRRDGKWLNVHYHCSGAPAAPLQ*

Supplementary figure 3. Numbering of exons with the corresponding amino acid sequence. When the codon of an amino acid is divided over two exons, the amino acid is placed in brackets. The linker domain contains exons 13-19 and is indicated by the yellow line. Note that exon 12, 18 and 20 contain different variations, depending on the splice variant, e.g. exon 12 ends on a valine when exon 13 is spliced in but ends on an alanine when exon 13 is spliced out.
