# Supplementary material for: Loss of CAMK2G affects intrinsic and motor behavior but has minimal impact on cognitive behavior
Source: Front Neurosci. 2023 Jan 6;16:1086994. doi: 10.3389/fnins.2022.1086994 (PMC9853378; doi:10.3389/fnins.2022.1086994)
Supplement: Supplementary file 4 [file Image_4.PDF]

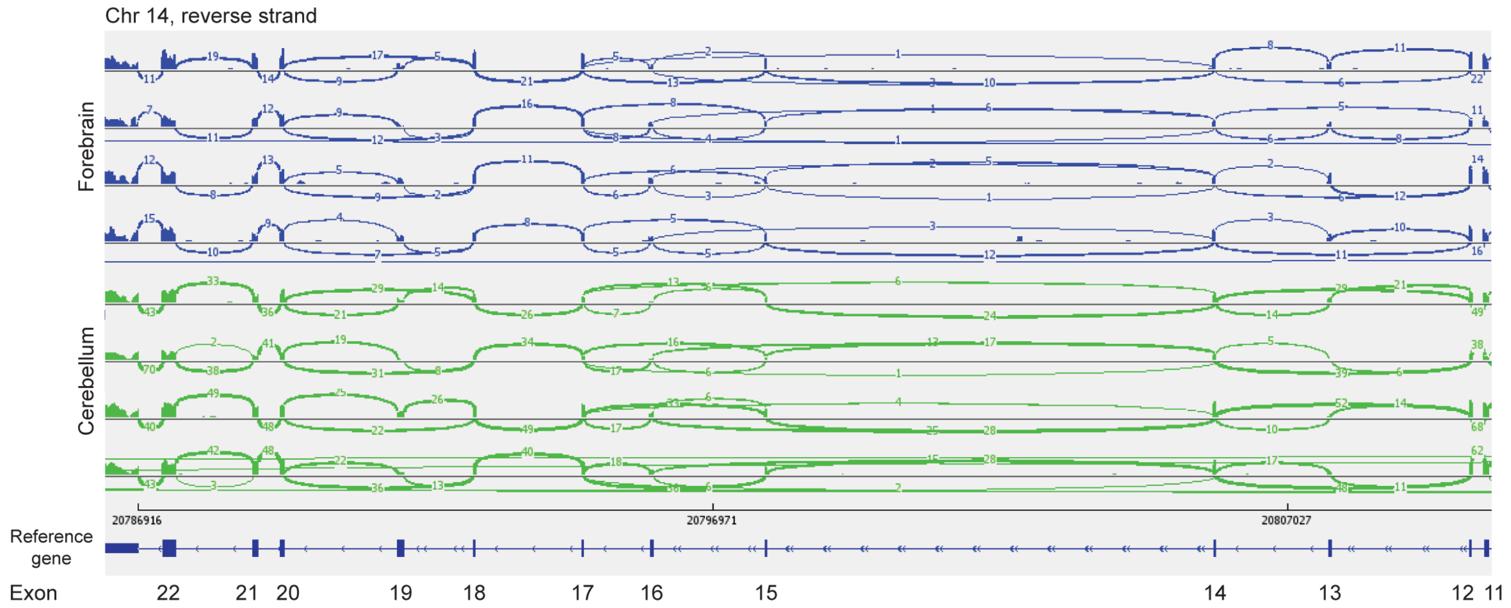

Supplementary Figure 4. Raw sashimi plots (IGV) showing amount of exon coverage and transcript reads between exons 11-23 of Camk2g in the forebrain (blue) and cerebellum (green) of the four mice that were analyzed. Camk2g is on the reverse strand, a reference gene with corresponding exon numbering is shown below.
