# Supplementary material for: Loss of CAMK2G affects intrinsic and motor behavior but has minimal impact on cognitive behavior
Source: Front Neurosci. 2023 Jan 6;16:1086994. doi: 10.3389/fnins.2022.1086994 (PMC9853378; doi:10.3389/fnins.2022.1086994)
Supplement: Supplementary file 5 [file Image_5.PDF]

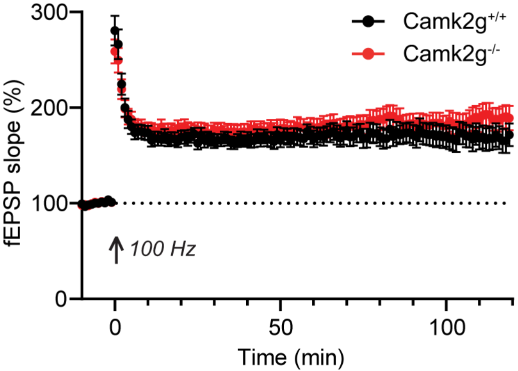

Supplementary Figure 5. Late-phase LTP did not differ between *Camk2g*$^{-/-}$ and *Camk2g*$^{+/+}$ mice (two-way repeated measures ANOVA on min 110-120, genotype: $F(1,48) = 1.56$, $p = 0.217$; n = 23/4 slices per mouse for *Camk2g*$^{+/+}$ and n = 31/6 for *Camk2g*$^{-/-}$). Data represents mean ± SEM.
